# The Relationship Between COVID-19 and the Development of Diabetic Ketoacidosis and New-Onset Type 1 Diabetes Mellitus

**DOI:** 10.7759/cureus.60711

**Published:** 2024-05-20

**Authors:** Creighton Kellogg, Louis C Haenel

**Affiliations:** 1 Medicine, Edward Via College of Osteopathic Medicine, Spartanburg, USA; 2 Endocrinology, Roper St. Francis Healthcare, Charleston, USA

**Keywords:** type 1 diabetes mellitus, autoimmune like, hyperlipidemia treatment, ketotic hyperglycemia, diabetic ketoacidosis (dka), covid-19

## Abstract

The COVID-19 pandemic with the outbreak of the severe acute respiratory syndrome coronavirus 2 (SARS-CoV-2) virus has been one of the largest topics of discussion in the medical world over the last few years. Most of the research has focused on the risks and correlation of chronic diseases and immunosuppression with the severity and mortality of the viral infection. Less research has occurred in the setting of post-infectious sequelae and the long-term effects of COVID-19 with the development of chronic conditions and diseases, such as new-onset type 1 diabetes mellitus. The incidence of diabetic ketoacidosis (DKA) has increased during the COVID-19 pandemic, but the relationship between the two conditions remains to be fully understood. We report the case of a 24-year-old male who presents with malaise, polyuria, polydipsia, headache, and fatigue and was eventually found to be in diabetic ketoacidosis (DKA). He had a history of COVID-19 infection 12 weeks prior to this presentation. He also had a family history of DKA and type 1 diabetes mellitus. This case highlights the need to perform an in-depth workup for each patient with DKA and new-onset diabetes mellitus in order to find a potential cause of the autoimmune condition.

## Introduction

Diabetic ketoacidosis (DKA) is an acute complication of diabetes characterized by hyperglycemia and ketoacidosis. Ketoacidosis is the factor of DKA that separates it from hyperglycemic hyperosmolar syndrome (HHS), which is another acute complication of diabetes mellitus. DKA usually occurs in younger patients with type 1 diabetes mellitus, while HHS occurs more in type 2 diabetes mellitus, but there can be a crossover. Clinical presentation can include polyuria, polydipsia, weight loss, abdominal pain, nausea, vomiting, and many others [1]. Initial workup for a patient with these findings concerning for DKA should include immediate evaluation that involves assessing vital signs, cardiorespiratory status, mental status, and volume status. Once the patient is stabilized and the labs indicate hyperglycemia, acidosis, the presence of ketones, and an elevated anion gap, the diagnosis of DKA can be made.

DKA can be the initial presentation of undiagnosed diabetes mellitus, or it may be an exacerbation due to inadequate management of the diabetes condition. In many cases, a contributing factor may be identified in the development of DKA. These can vary from illnesses such as infection, myocardial infarction, stroke, or acute pancreatitis, to drugs such as sodium-glucose co-transporter 2 (SGLT2) inhibitors, clozapine, lithium, and cocaine abuse [2]. Infections can lead to DKA by causing the body to release higher levels of cortisol and catecholamines, which leads to a hormonal imbalance resulting in increased hepatic gluconeogenesis, glycogenolysis, and lipolysis [3]. The release of free fatty acids from lipolysis into the blood is followed by oxidation of these free fatty acids into ketone bodies such as beta-hydroxybutyrate and acetoacetate [4]. These ketones in the blood create a high anion gap metabolic acidosis with compensatory hyperventilation in order to reduce the systemic carbon dioxide. In recent years, reports have shown that infections have been a common precipitating factor in the setting of new-onset diabetes. With this case report, it has been shown that COVID-19 may be one of these infections as it can serve as a potential initiating factor of DKA. The purpose of this case report is to show that prior infections, including COVID-19, may serve as the catalyst for development of DKA and new-onset type 1 diabetes mellitus and should therefore cause physicians to ask about recent infections in those with newly acquired type 1 diabetes mellitus.

## Case presentation

A 24-year-old male with no past medical history presented to the urgent care with complaints of malaise for three weeks, persistent polyuria with nocturia, polydipsia, headache, and fatigue. The patient later mentioned that he had weighed as much as 150 pounds, but he weighed 113 pounds with a BMI of 17.3 at that time. The timeframe for the weight loss was unknown to the patient. The patient was taking no medications or supplements, and he was living a healthy lifestyle that included a regular exercise program. Family history showed that his father was recently diagnosed with type 1 diabetes mellitus during his 50s. Other pertinent history included a COVID-19 infection three months prior to this presentation, but no other recent illnesses. Upon arrival to the urgent care, a point-of-care urinalysis and basic metabolic panel were performed which showed glucosuria, large urinary ketones, hyponatremia, hyperglycemia, and an elevated anion gap (Table 1). His vital signs included a temperature of 37.2 degrees Celsius, blood pressure of 110/68, and heart rate of 78, and a physical exam was unremarkable. The urgent care recommended transfer to the emergency department for further workup.

**Table 1:** Initial basic metabolic panel performed at the urgent care.

Lab Value	Patient's Value	Reference Range [5]
Sodium (Na)	125 mmol/L	136-145 mmol/L
Fasting glucose	545 mg/dL	70-99 mg/dL
Anion gap	15 mmol/L	7-13 mmol/L

Once the patient arrived in the emergency department, he was hemodynamically stable. Repeat labs showed hyponatremia, hyperglycemia, positive serum ketones, an elevated anion gap, and an acidosis on venous blood gas. Other lab findings included hypokalemia, hypochloremia, and a decreased CO_2_ (Table 2). The ED diagnosed this patient with diabetic ketoacidosis (DKA) without coma and new-onset diabetes mellitus, likely type 1. Other diagnoses included pseudohyponatremia (corrected for glucose level), hyperlipidemia with hypertriglyceridemia, hypokalemia, and hypocalcemia.

**Table 2:** Repeat basic metabolic panel performed in the emergency department.

Lab Value	Patient's Value	Reference Range [5]
Sodium (Na)	120 mmol/L	136-145 mmol/L
Potassium (K)	3.1 mmol/L	3.5-5.0 mmol/L
Chloride (Cl)	83 mmol/L	98-106 mmol/L
CO_2_	7 mmol/L	23-30 mmol/L
Blood urea nitrogen (BUN)	7 mg/dL	8-20 mg/dL
Creatinine	0.5 mg/dL	0.7-1.3 mg/dL
Fasting glucose	380 mg/dL	70-99 mg/dL
Anion gap	24 mmol/L	7-13 mmol/L
pH	7.2	7.35-7.45

The patient was started on an insulin drip with DKA protocol while in the emergency department with continuous IV fluids, IV potassium, and was kept nothing by mouth (NPO). Endocrinology recommended to stay on insulin drip until the metabolic acidosis cleared, which would result in the eventual transition to a basal-bolus insulin regimen.

Over the next few days of hospitalization, the patient experienced no significant symptoms or metabolic derangements. The majority of his hospital course was unremarkable and involved correction of electrolytes with continuous glucose checks. The patient’s hemoglobin A1c came back at 10.5%, indicating a diagnosis of diabetes mellitus. The patient’s triglyceride level was severely elevated, and endocrinology recommended continued insulin drip throughout his week-long hospital course. Other lipid levels at this time showed elevated total cholesterol, decreased high-density lipoprotein (HDL), elevated very low-density lipoprotein (VLDL), and a normal value for low-density lipoprotein (LDL) (Table 3). He was started on 80 mg of atorvastatin and 48 mg of fenofibrate. The patient was discharged home on the seventh day of hospitalization.

**Table 3:** In-hospital hemoglobin A1c value and lipid panel.

Lab Value	Patient's Value	Reference Range [5]
Hemoglobin A1c	10.5%	4.0%-5.6%
Triglycerides	1918 mg/dL	<150 mg/dL
Total cholesterol	688 mg/dL	<200 mg/dL
High-density lipoprotein (HDL)	16 mg/dL	>60 mg/dL
Very low-density lipoprotein (VLDL)	115 mg/dL	<30 mg/dL
Low-density lipoprotein (LDL)	25 mg/dL	<100 mg/dL

Three weeks after the initial presentation, the patient was seen in the endocrinology office for follow-up. The patient remained steady with his insulin requirements on a basal-bolus regimen, and his glucose monitoring was consistent. Repeat hemoglobin A1c showed a decrease down to 7.4%. The patient was asymptomatic, and vital signs and physical exam were unremarkable. Repeat lipid levels showed an improved lipid panel with a decreased total cholesterol, triglycerides, LDL, and an increase in HDL (Table 4). The patient was advised to continue his lipid-lowering management plan. He will also be considered for genetic testing considering his elevated lipid levels and family history. The patient will remain in close follow-up with endocrinology, dietitian, and a diabetic educator.

**Table 4:** Repeat Hemoglobin A1c and lipid panel during outpatient follow-up.

Lab Value	Patient's Value	Reference Range [5]
Hemoglobin A1c	7.40%	4.0-5.6%
Triglycerides	125 mg/dL	<150 mg/dL
Total cholesterol	167 mg/dL	<200 mg/dL
High-density lipoprotein (HDL)	51 mg/dL	>60 mg/dL
Low-density lipoprotein (LDL)	91 mg/dL	<100 mg/dL

## Discussion

The COVID-19 pandemic with the outbreak of severe acute respiratory syndrome coronavirus 2 (SARS-CoV-2) and diabetes have been studied to define a relationship between the two diseases. It has been determined that diabetes is a risk factor for worse outcomes in those who contract the COVID-19 infection, including higher hospital admission rates, development of severe pneumonia, and higher mortality rates [6]. In addition, it has also been hypothesized that the COVID-19 infection can precipitate DKA. Two different explanations have been theorized. The first explanation involves the interaction between the SARS-CoV-2 virus and the angiotensin-converting enzyme 2 (ACE2) [7]. ACE2 is the functional receptor of the SARS-CoV-2 virus, and this receptor is also found in pancreatic islet cells. The function of this receptor in the pancreas is degrading angiotensin 2, which causes decreased proliferation and induces local inflammation within the pancreatic islets [7]. Therefore, the ACE2 receptor serves a protective role in the pancreas. With the SARS-CoV-2 virus, there is decreased expression of ACE2, resulting in elevated levels of angiotensin 2, resulting in long-term deleterious effects on the pancreas and the islet cells. The second explanation of the COVID-19 infection precipitating DKA hypothesizes that the virus can directly bind to the pancreatic islet cells and cause direct damage [8]. This results in pancreatic cell modification, hyperglycemia, and acute diabetes in those with severe SARS infections. Some studies show that the virus directly enters the islet cells via the ACE2 receptor, while others explain that the virus triggers a release of acute proinflammatory cytokines that can directly damage islet cells [9]. Either way, results have shown that patients who have suffered from SARS-CoV-2 infection are at an increased risk of developing new-onset diabetes in addition to the development of DKA.

Our patient is fascinating in the fact that there was a long delay between the COVID-19 infection and the development of DKA. It is also interesting how this patient had a family history of type 1 diabetes in his father who also initially presented in DKA. This case brings up the fascinating question of whether the COVID-19 infection was the sole reason for developing DKA, or if this patient’s genetic predisposition was the major contributing factor for developing type 1 diabetes. Meta-analyses conducted during the past three years in response to the COVID-19 pandemic have shown that patients who suffer from a SARS-CoV-2 infection are at an elevated risk of developing DKA and new-onset type 1 diabetes mellitus as compared to non-COVID-19 control groups [10]. This specific meta-analysis also discovered that development of DKA following the viral infection occurs at a higher rate in the United States as compared to European countries [10]. In another meta-analysis involving over 124,000 children, it has been shown that the incidence of DKA in children significantly increased during the COVID-19 pandemic [11]. This study also highlights that the severity of DKA can be correlated to a history of prior COVID-19 infection [11]. Based on the increased incidence of DKA in the setting of previously having COVID-19, it is likely that the history of a previous COVID-19 infection in this patient played a role in the development of his DKA. The combination of genetic predisposition and COVID-19 infection in this patient could have potentially expedited the development of pancreatic islet cell destruction and eventual insulin deficiency.

## Conclusions

It is well known that patients who suffer from chronic diseases and immunosuppression and then contract the SARS-CoV-2 infection have a more serious infection with higher rates of morbidity and mortality as compared to healthy individuals. However, fewer studies have investigated the possibility of the virus increasing the risk of long-term chronic diseases and comorbidities, such as new-onset type 1 diabetes mellitus. The pathogenesis of this connection remains in debate and involves hypotheses of the virus directly attacking pancreatic islet cells through its interaction with the ACE2 receptor. In this case, the additional intriguing factor was this patient having a family history of type 1 diabetes mellitus, increasing his chances of acquiring this condition. This case indicates the potential relationship between COVID-19 and DKA in the setting of new-onset type 1 diabetes mellitus, and it also highlights the need for physicians and practitioners to be aware of the potential development of long-term conditions and diseases following SARS-CoV-2 infection resolution. In addition, each patient who is diagnosed with DKA should undergo further evaluation and workup for potential causes of the illness and new-onset type 1 diabetes mellitus.
